# P-71. Combined Orthopaedic and Infectious Disease Periprosthetic Joint Infection Clinic: A Survey Study

**DOI:** 10.1093/ofid/ofae631.278

**Published:** 2025-01-29

**Authors:** Eric Dilbone, Justin Leal, Edward F Hendershot, Erin Gettler, Jessica Seidelman, Thorsten M Seyler, William A Jiranek

**Affiliations:** Duke University Hospital, Durham, North Carolina; Duke University Health, Durham, North Carolina; Duke University, Durham, North Carolina; Duke University Medical Center, Durham, NC; Duke University School of Medicine, Durham, North Carolina; Duke University, Durham, North Carolina; Duke University, Durham, North Carolina

## Abstract

**Background:**

The rising incidence of total joint arthroplasties being performed is leading to an increased number of prosthetic joint infections (PJI). PJI treatment is complex, thus a collaborative approach involving orthopaedic surgeons and infectious disease (ID) providers is growing. This single institution has a weekly combined Ortho-ID (OID) clinic that sees PJI patients and is staffed by joint replacement fellows and ID attendings. This project sought to survey the joint replacement fellow participants from recent years regarding the impact of this experience on their subsequent clinical practice.

Figure 1

**Figure:**
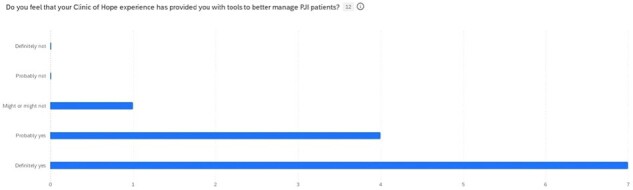

**Methods:**

A survey was distributed to fellows who participated in the combined OID clinic during their single year of fellowship from 2020 through 2023 to evaluate the impact of this experience on their development as clinicians and current clinical practice. An anonymous survey included all current and former fellows since the inception of this clinic in 2020. Institutional review board (IRB) approval was obtained prior to the distribution of the survey.

Table 1

**Figure:**
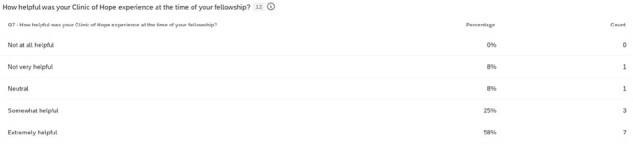

**Results:**

A total of 14 current and former fellows received the survey and 12 responded. The majority of the respondents are currently in an academic practice setting (75%), with the rest being hospital employed (17%) or private practice (8%). Taking care of PJI patients makes up anywhere from 0-40% of the respondents’ practices. 92% of former fellows felt that the multi-disciplinary approach to this clinic helped prepare them for managing PJI patients in practice today. 92% also felt that this experience would be beneficial going forward for future fellows and that the current model of ½ day clinic once per month was sufficient.

**Conclusion:**

A combined OID clinic is increasingly a popular approach to the management of PJI patients, but little is known about the utility of this clinic setting for trainees. This survey of participants in this model of clinic shows that once in practice, many orthopedic surgeons found that this experience gave them tools to better manage their PJI patients. Arthroplasty fellowship programs should consider including a combined OID clinic as part of their fellowship.

**Disclosures:**

**Jessica Seidelman, MD, MPH**, 3M: Expert Testimony

